# Study of cattle microbiota in different regions of Kazakhstan using 16S metabarcoding analysis

**DOI:** 10.1038/s41598-022-20732-4

**Published:** 2022-09-30

**Authors:** Aida Daugaliyeva, Saule Daugaliyeva, Alexander Ashanin, Serik Kanatbayev, Chiara Beltramo, Simone Peletto

**Affiliations:** 1 Kazakh Research Institute for Livestock and Fodder Production, St. Zhandosova 51, Almaty, Kazakhstan; 2 Research and Production Center for Microbiology and Virology, St. Bogenbai batyr 105, Almaty, Kazakhstan; 3 West Kazakhstan Research Veterinary Station, St. Gagarina 52/1, Uralsk, Kazakhstan; 4 Istituto Zooprofilattico Sperimentale del Piemonte, Liguria E Valle d’Aosta, via Bologna 148, 10154 Turin, Italy

**Keywords:** Ecology, Microbiology, Zoology

## Abstract

Methane (CH_4_) is an important greenhouse gas (GHG). Enteric methane emissions from farmed ruminant livestock account for approximately 15% of global GHG emissions, with approximately 44% of livestock emissions in the form of methane. The purpose of the research is to study the influence of feeding types and regional characteristics of Kazakhstan on the microbiota of feces and the number of methane-forming archaea of beef and meat-and-dairy cattle productivity. For this purpose, fecal samples were taken rectally from 37 cattle heads from four regions of Kazakhstan (Western, Southern, Northern and Southeast). The taxonomic composition of the community in all samples was determined by 16S metabarcoding; additionally alpha and beta diversities were calculated. The dominant phyla were: *Firmicutes* (57.30%), *Bacteroidetes* (17.00%), *Verrucomicrobia* (6.88%), *Euryarchaeota* (6.49%), *Actinobacteria* (4.77%) and *Patescibacteria* (3.38%). Significant differences with regard to methanogens bacteria were found: *Euryarchaeota* were less present in animals from Western Kazakhstan (2.40%), while *Methanobacteriales* and *Methanobrevibacter* were prevalent in Southeast, and less abundant in Western region. Western Kazakhstan differs from the other regions likely because animals are mainly grazed in the pasture. Thus, grazing animals has an impact on their microbiota thus leading to a decrease in methane emissions.

## Introduction

Methane (CH_4_) is an important greenhouse gas (GHG). Enteric methane emissions from farmed ruminant livestock account for approximately 15% of global GHG emissions^1^, with approximately 44% of livestock emissions in the form of methane^2^. Most methane is produced in the rumen (87%) and a small amount (13%) is produced in the large intestine^1^. The rumen microbiome is a complex community of prokaryotes, eukaryotes, and viruses. The prokaryotes include bacteria, primarily anaerobic bacteria, and archaea^3^. Rumen archaea are strictly anaerobic and are the only known microorganisms present in the rumen capable of producing methane^4,5^. Such archaea are referred to as methanogens. Archaea are found in the rumen in the range of 10^6^ to 10^8^ cells per ml, accounting for less than 4% of the microbial community^5^.

According to a meta-analysis of worldwide data, 90% of rumen methanogens belong to the following genera^5,6^; hydrogenotrophic *Methanobrevibacter spp*. are the main methanogens making up 75–78% of the methanogenic archaea in rumen^7,8^, *Methanomicrobium* (7.7% of the methanogen population, *Methanosphaera* (9.8%), "Rum Cluster C", now referred to as *Thermoplasma* (7.4%) and *Methanobacterium* (1.2%). Most methanogens remove hydrogen by reducing CO_2_ with hydrogen gas to form methane. In contrast, *Methanosphaera stadtmanae* only produces methane through the reduction of methanol with H_2_, having one of the most stringent energy metabolisms of any methanogenic archaea. Methane production maintains a low hydrogen concentration in the rumen, which allows methanogenic bacteria to promote the growth of other types of bacteria, thus resulting in a more efficient fermentation^5^. However, the methane produced in the rumen is regurgitated, resulting in air pollution.

*Methanobacteriales* and *Methanomassiliicoccales* are the main orders and account for up to 99.98% of the methanogen community in rumen samples^7,8^.

Methane is a powerful greenhouse gas that cannot be absorbed by livestock and is released into the environment, contributing to climate change.

It should also be noted that methane production by archaea represents an energy loss of about 2–12% of gross energy intake^5,9^, meaning this energy is no longer available for animal growth, lactation, maintenance or pregnancy. Lowering emissions methane would benefit both the environment and the efficiency of livestock production^10^.

Kazakhstan covers an area equal to 2724.9 thousand square meters. km. (1048.3 thousand sq. miles) and is in ninth place in the world in terms of territory. Due to the vast territory of the country, the climate in different regions is very different. Due to the huge differences in climate, soil, flora in different regions of the country, the conditions for keeping and feeding cattle vary significantly. On these basis, animals from different regions of Kazakhstan were taken into our experiment.

The purpose of the research is to study the influence of feeding types and regional characteristics of Kazakhstan on the microbiota of feces and the number of methane-forming archaea of beef and meat-and-dairy cattle productivity.

## Materials and methods

In the selected farms, the fodder base, the chemical composition and nutritional value of fodder, the type of feeding, the structure of the ration, the breed of animals, their live weight and productivity were studied, and fecal samples were taken.

The use of animals, including experimental procedures, and collection of the fecal samples, was approval by the Commission on Bioethics of the Kazakh Research Institute of Livestock and Fodder Production in accordance with the requirements of the provisions of the "European Convention for the Protection of Vertebrate Animals used for Experimental and Other Scientific Purposes" and the provisions Guide for Care and Use of Laboratory Animals.

Fecal samples were taken rectally from 37 cattle and beef of dual purpose (beef-dairy) production from four regions of Kazakhstan: (Western (3 farms), Southern (2 farms), Northern (2 farms) and Southeastern (5 farms). Fecal samples were collected in a clean labeled container. Sealed sterile polypropylene sample containers were immediately frozen on dry ice. All samples were stored in the laboratory at -80 °C until analysis.

### DNA extraction, PCR amplification and sequencing on MiSeq Illumina

Two hundred milligrams of feces samples were taken for DNA isolation. DNA from the samples was isolated using the PureLink Microbiome DNA Purification Kit (Invitrogen, Carlsbad, USA) according to the manufacturer's protocol. The DNA concentration was measured on a Qubit 2.0 fluorimeter using a Qubit dsDNA HS Assay Kit (Life Technologies, Oregon, USA).

The genetic libraries were prepared for sequencing following the 16S Metagenomic Sequencing Library Preparation guide (part no. 15044223 rev. A).

PCR amplification was carried out immediately after DNA extraction, using primers targeting the V3 and V4 hypervariable regions of the 16S rRNA. For amplification were used universal bacterial primers with the addition of the Illumina adapters, and contained the following sequences of the nucleotide pairs: 5′-TCGTCGGCAGCGTCAGATGTGTATAAGAGACAGCCTACGGGNGGWGCAG-3′ for the forward primer, and 5′-GTCTCGTGGGCTCGGAGATGTGTATAAGAGACAGGACTACHVGGGTATCTAATCC-3′ for the reverse primer^11^. Each DNA sample was amplified using the KAPA HiFi Hot Star Ready Mix (KAPA Biosystems, Cape Town, South Africa).

The reaction mixture consisted of 2.5 μl of the DNA template, 5 µl of each primer in the concentration of 1 µM, and 12.5 µl of KAPA HiFi Hot Start Ready Mix in the 2X concentration to a final volume of 25 μL. The amplification cycles were performed according to the following program: initial denaturation at 95 °C for three minutes, followed by 25 amplification cycles at 95 °C for 30 s, primer annealing at 55 °C for 30 s, and extension at 72 °C for 30 s, and with a final elongation at 72 °C for five minutes. The PCR amplification was made in an Eppendorf Master ProS thermal cycler (Eppendorf, Hamburg, Germany). The PCR product was purified using an Agencourt AMPure PCR purification kit (Beckman Coulter. Beverly, Massachusetts, USA). Nextera XT Index primer adapters (Illumina, San Diego, USA) were added to each sample through the amplification in the reaction mixture containing: 12.5 µl of KAPA HiFi Hot Start Ready Mix, 2,5 µl of each index primer, 2,5 µl of the PCR product and 5 µl of high purity water for PCR. The amplification program included: one cycle at 95 °C for three minutes, followed by eight cycles of amplification at 95 °C for 30 s, one cycle at 55 °C for 30 s, one cycle at 72 °C for 30 s, and one cycle at 72 °C for five minutes. The PCR product with added indices was also purified using an Agencourt AMPure PCR purification kit. At each stage of preparing the libraries after the amplification, the concentration and the size of the obtained PCR products were determined through their detection in the agarose gel and on an Agilent 2100 Bioanalyzer (Waldbronn, Germany) using an Agilent DNA 1000 Kit (Agilent Technologies, Germany), quantified using Qubit dsDNA HS Assay Kit (Life Technologies, Oregon, USA). There was no amplification product observed in the negative extraction controls. Each sample was diluted to a concentration of 4 nM and combined into a single pool. The pooled libraries were denatured with NaOH and diluted with the hybridization buffer. PhiX control was added to the pooled library in the amount of 15% of the volume. The pooled libraries whith PhiX were sequenced on an Illumina MiSeq device using a 600-cycle MiSeq Reagent Kit v3 (Illumina, San Diego, USA), following the manufacturer's recommendations.

### Bioinformatics and data analysis

The data generated by the massive sequencing were analyzed with the CLC Genomics Workbench software (Qiagen). Specific tools for OTU (Operational Taxonomic Unit) clustering analysis available in the CLC Microbial Genomics Module package were used. In short, the reads were filtered by quality, grouped into OTUs and classified using the SILVA database to create an OTU table. Alpha diversity (diversity within groups using the species richness index and Shannon index) and beta diversity (diversity between groups using the Bray–Curtis index and Principal Coordinates Analysis, PcoA) were assessed considering the samples according to their geographical origin (i.e. Kazakh region). Statistical significance for alpha and beta diversities was determined by Kruskal-Walles and Permanova analysis, together with a Differential abundance analysis, considering a p-value of 0.05. Finally, a heat map was created to look for links between geographical origin and microbiota composition by phylum.

### Ethical approval

Ethics approval and consent to participate. The experiment was carried out after approval by the Commission on Bioethics of the Kazakh Research Institute of Livestock and Fodder Production.

## Results

### Comparative characteristics of rations for feeding cattle from different regions of the Republic of Kazakhstan and the impact of animal feeding types on the faecal microbiota

Due to the huge differences in the natural and climatic conditions of Kazakhstan, animals from different regions of Kazakhstan were enrolled for this study. The difference in soil and climatic conditions of different zones has a significant impact on the type of feeding (Table 1) and the composition of diets, which has a certain effect on the microbiota of intestinal contents and methanogenic archaea in particular.

**Table 1:** Animal diets in different regions of Kazakhstan.

Name of animals	Name of the farm	Age and sex group of animals	Breed	Feeding type	Sampling time
**North-Kazakhstan region**					
1. Average: 5AnC,6AnC,7AnC	LLP "Bereke-Akzhar"	Heifers	Aberdeen Angus	Hay concentrate	May
2. Average:1BelC, 4BelC	LLP "Alabota"	Cows	Kazakh white-headed	Haylage-hay-concentrate (haylage (oats, barley, wheat 30% each)—18 kg, hay—8 kg, concentrates—3 kg (barley, wheat))	May
**Almaty (Southeast) region**					
3. Average:1Ba, 2Ba,3Ba	LLP «Bayserke-Agro»	Gobies	Kazakh white-headed	Silage concentrate (corn silage 45%, mixed grass hay 11%, crushed barley with chaff 44%)	June
4. Average:1An,2An, 3An	Farm «Bimuratov»	Cows	Aberdeen Angus	Hay concentrate (haylage −7 kg, corn feed—3 kg, hay −10 kg)	May
5. Average:Tv1, Tv2, Tv3	Farm «Akylbay»	Cows	Local livestock	Herb pasture	May
6. Average:1Ki-1, 2Ki, 3Ki	Farm «Nadirov»	Milking cows	Alatau	Pasture + concentrates (wormwood, forbs, alfalfa) + concentrates (bran with barley—3–4 kg)	June
7. Average:4By, 5Ki-1, 6Ki-1	Farm «Nadirov»	Dry cows	Alatau	Pasture + concentrates (wormwood, forbs, alfalfa) + concentrates (bran with barley—3–4 kg)	June
2Kr KB	EPF «Kurozek»	Gobies	Kazakh white-headed	Hay concentrate (hay, straw and grain fodder)	April
3Kr Al	EPF «Kurozek»	Gobies	Alatau	Hay concentrate (hay, straw and grain fodder)	April
**West-Kazakhstan region**					
10. Average:4Z, 5Z, 6Z	Farm "Shunaibekov"	Cows	Kazakh white-headed	Pasture (feather grass, sage, volost)	May
11. Average: 1Ger, 2Ger,3Ger	Farm "Shovda"	Cows	Hereford	Pasture (feather grass, tansy, sage)	May
12. Average:1Z,2Z, 3Z	Farm «Shibat»	Cows	Aberdeen Angus	Pasture (wormwood, feather grass)	May
**Turkestan (South) region**					
13. Average:1КB Ch, 2КB Ch, 3 KB Ch	APC «Et Ark Manket»	Gobies	Kazakh white-headed	Hay concentrate (Wheat 1 kg, Barley 1 kg, Corn 0.5 kg, Meal 1 kg, Compound feed 2 kg, "Kormovik" vitamin supplement 0.1 kg, Alfalfa 1 kg, Straw 1 kg)	April
14. Average:2An Ch, 3An Ch, 4An Ch	APC «Et Ark Manket»	Gobies	Aberdeen Angus	Hay concentrate (Wheat 1 kg, Barley 1 kg, Corn 0.5 kg, Meal 1 kg, Compound Feed 2 kg, Vitamin supplement "Kormovik" 0.1 kg, Alfalfa 1 kg, Straw 1 kg)	April

In the course of the research work, regions and specific agricultural formations were identified in the context of these regions.

In North Kazakhstan, the fodder base is represented by such fodders as alfalfa hay, herb hay, alfalfa haylage, wheat straw, fodder wheat and sunflower cake. The feed is mainly of 2 quality classes. The live weight of cattle ranged from 375 to 480 kg. Feeding type: hay-concentrate and haylage-hay-concentrate.

In the Western region, the animals were on the pasture, represented by the green mass of feather grass, hair, sage and tansy. Beef cattle are represented by the following breeds: Kazakh white-headed, Aberdeen-Angus and Hereford. Average live weight is 350–550 kg.

In the Southeast region, the fodder base consists of wheat hay, sainfoin + alfalfa hay, mountain hay, herb haylage, corn silage and crushed corn. The feed is mainly of 2 and 3 classes. Hay-concentrate type of feeding is used, as well as pastures. Livestock of Angus, Kazakh white-headed breeds and animals of the local population are kept. Live weight of young animals is in the range of 360–380 kg.

The diets of the Southern Region include natural grass hay, alfalfa hay, wheat straw, alfalfa haylage and concentrates. Hay-concentrate type of livestock feeding is widespread in the region. The average live weight of bulls for fattening of the Kazakh white-headed and Angus breeds—360–420 kg with a daily increase in live weight of 870–920 g.

The composition of the fecal microbiota depending on the type of feeding is presented in Table 2.

**Table 2:** The content of methanogenic archaea in feces.

Feeding type	Fecal microbiota, %	
	Bacteria	Archaea
Hay—concentrated	96.29 ± 12.7	3.37 ± 1.46
Haylage—hay—concentrated	96.05 ± 27.16	3.63 ± 1.04
Haylage—concentrated	93.8 ± 12.41	6.01 ± 2.29
Pasture	97.61 ± 0.49	1.32 ± 0.24
Pasture—concentrated	93.24 ± 3.73	4.87 ± 1.29
Silage—concentrated	98.59 ± 13.0	1.54 ± 0.5
Concentrated	97.03 ± 3.88	2.78 ± 0.22

From the data of Table 2 it follows that the largest amount of *Bacteria* was found in the faeces of animals with silage-concentrated feeding (98.59 ± 13.0%), and the smallest—with pasture-concentrated (93.24 ± 3.73%) and haylage—concentrated (93.8 ± 12.41%) types of feeding. The differences amounted to 5.35 and 4.79 absolute percent, respectively. However, the differences were not significant at P < 0.95. For other types of feeding, the content was almost the same and ranged from 96.05 ± 27.16%—97.03 ± 3.88% and is also not reliable (P < 0.95).

The content of methanogenic microbes in faeces also largely depends on the type of animal feeding. Thus, the smallest number of them was found when keeping animals on pasture (1.32 ± 0.24%) and silage-concentrated type of feeding (1.54 ± 0.5%), and the largest—with haylage-concentrated type of feeding (6.01 ± 2.29%). Differences were significant at the level of P > 0.95.

With hay-concentrate and haylage-hay-concentrate types of feeding, the content of methanogenic microbes in comparison with pasture content was higher by 2.05 and 2.31 absolute percent, respectively, or more by 2.55 and 2.75 times. However, the differences were not significant (P < 0.95).

Certain differences are observed between hay-concentrate and silo-concentrate types of feeding. The difference between them in terms of the content of methanogenic microbes was 1.83 absolute percent in favor of the latter. Similar data were obtained in studies by Uprety D.C., Subash Dhar, Dong Hongmin et al.^12^. The authors argue that the formation of methane in the silo-concentrate type of feeding was 1.25 times lower compared to the hay-concentrated diet (283 L versus 353 L per head per day).

A similar pattern is observed with pasture-concentrated and concentrated types of feeding. Thus, in the feces of these animals, the content of methanogenic microbes was 4.87 ± 1.29% and 2.78 ± 0.22% or higher by 3.55 (P > 0.95) and 1.46 (P < 0.95) absolute percent compared to the pasture type of feeding.

Thus, the highest content of Bacteria in the faeces of animals was found with the silo-concentrate type of feeding, and the smallest amount of Arhaea was noted with the pasture type of feeding.

### Fecal bacterial communities from different regions of Kazakhstan in beef and dual-purpose cattle

**Figure 1:**
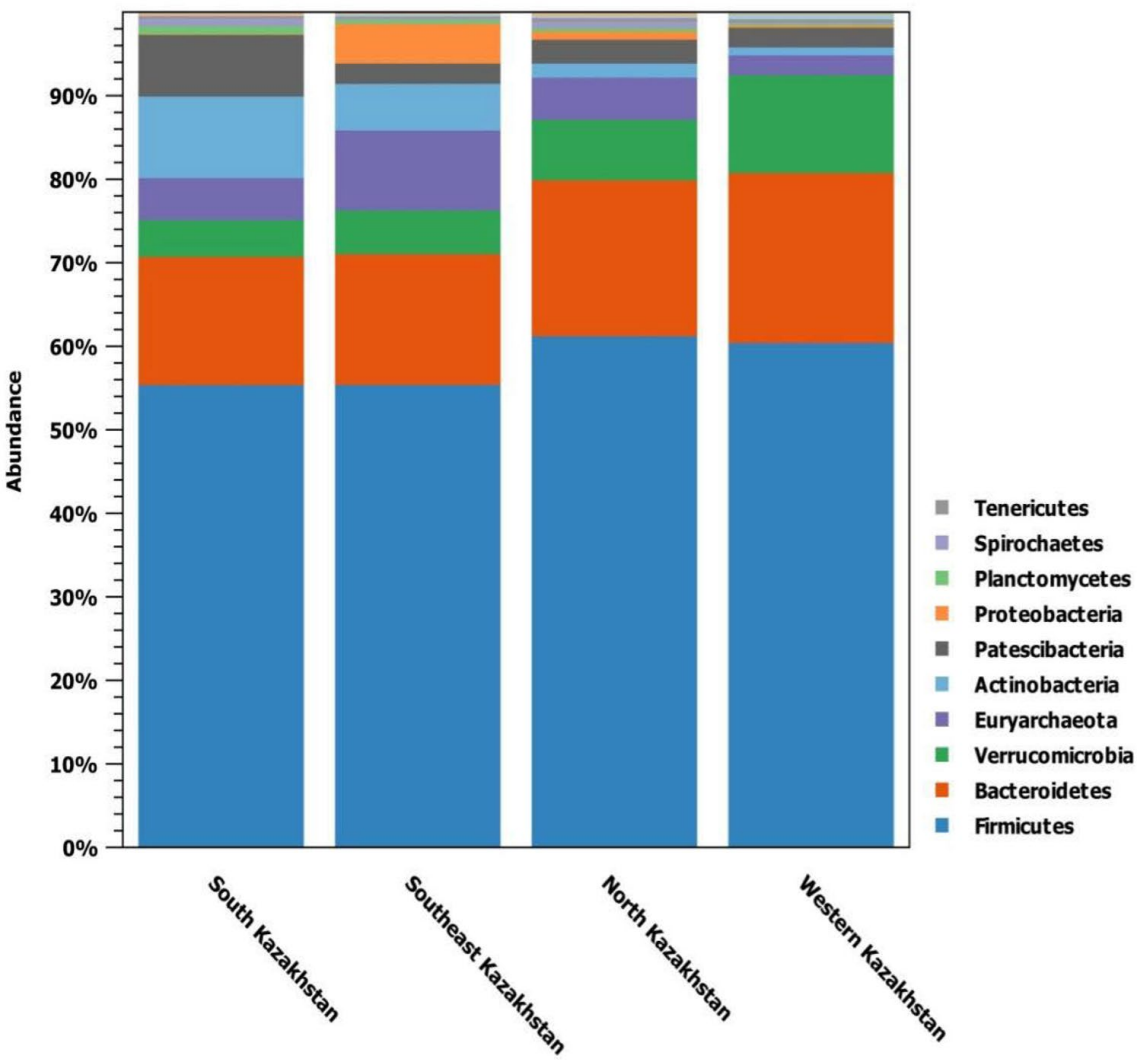
Relative abundance of fecal bacterial communities at the phylum level. The legend reports the 10 most abundant phyla.

Microbial composition of the feces collected from the rectum of cattle was examined based on the OTU table generated by the CLC Genomics Workbench software using the SILVA database as a reference. Samples were grouped according to the geographical origin (i.e. Western, Southern, Northern and Southeast regions of Kazakhstan) for analysis. In total, 22 microbial phyla were identified. Figure 1 shows phyla distribution by Kazakh regions.

The most abundant phylum was *Firmicutes*, ranging from 55.5% to 61.3% of relative abundance percentages, followed by *Bacteroidetes* (15.3–20.4%), *Verrucomicrobia* (4.4–11.7%) and *Euryarchaeota* (5.0–9.6%).

Firmicutes resulted to be widespread in cattle microbiota from all regions of Kazakhstan with non significant fluctuations. So, in the Northern region, their relative amount was 61.3%, in the Western 60.5%, in the Southern and Southeast 55.5%. The phylum of *Bacteroidetes* was next in terms of prevalence: Northern region (18.7%), Western (20.4%), Southern (15.3%), and Southeast (15.7%). The minimum amount of *Euryarchaeota* methanogens was found in animals of the Western region (2.4%), and the maximum in animals of the Southeast region (9.6%).

### Fecal methanogens from different regions of Kazakhstan in beef and dual-purpose cattle

**Figure 2:**
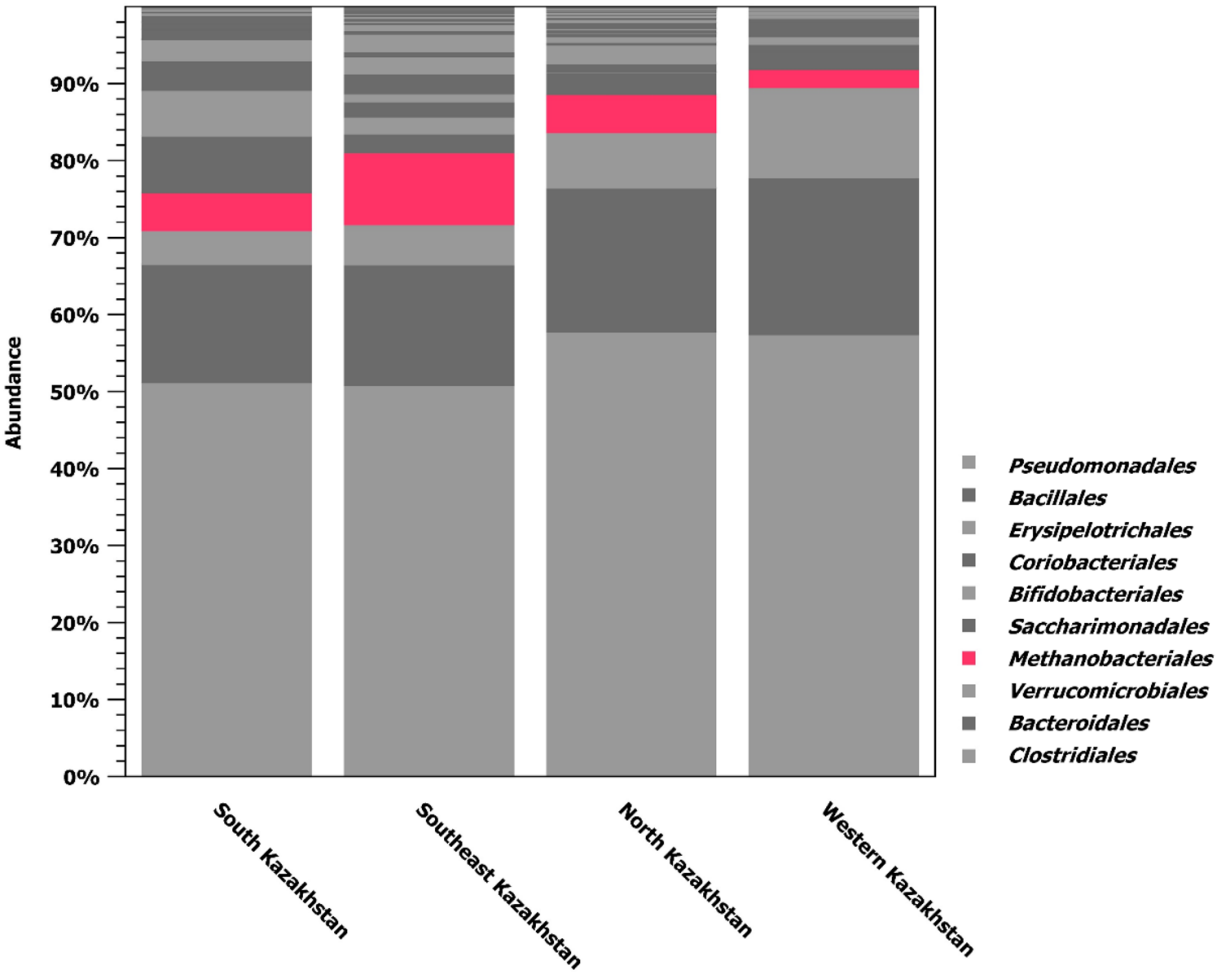
Relative abundance of fecal bacteria communities at an order level. *Methanobacteriales* are highlighted in pink-red. The legend reports the 10 most abundant orders.

The relative number of fecal bacterial communities at the order level is shown in Fig. 2.

Methane-forming archaea were mainly represented by the order *Methanobacteriales* (Fig. 2). Most of *Methanobacteriales* were detected in samples from Southeast Kazakhstan, while they were less present in Western Kazakhstan. The prevalence of other methanogenic groups was negligible.

**Figure 3:**
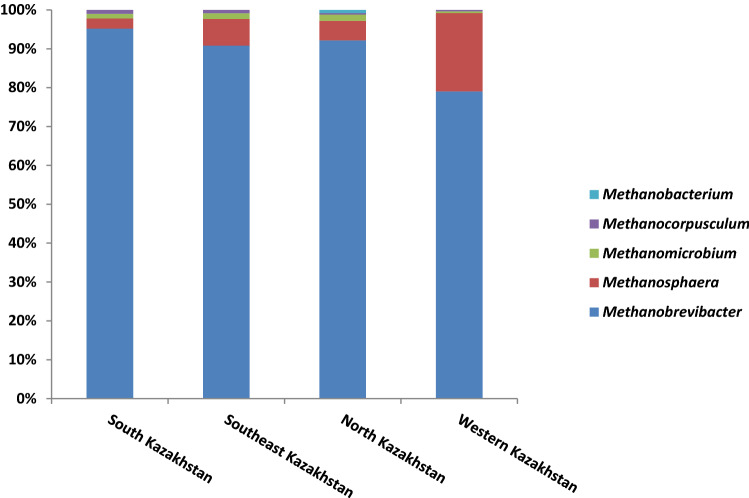
Relative abundance of methanogens at the genus level in animal samples from different regions of Kazakhstan.

Relative abundance of methanogens at the genus level as distributed for each Kazakh region is shown in Fig. 3.

*Methanobrevibacter* was the most abundant genus among methanogens. The highest amount was found in the Southeast region (8.6%) and the lowest in the Western region (1.8%). This was followed by the genus *Methanosphaera* with a prevalence falling in the range 0.13–0.65%. Other methanogens genera were detected in trace amounts.

### Heat-map of microbial community composition

**Figure 4:**
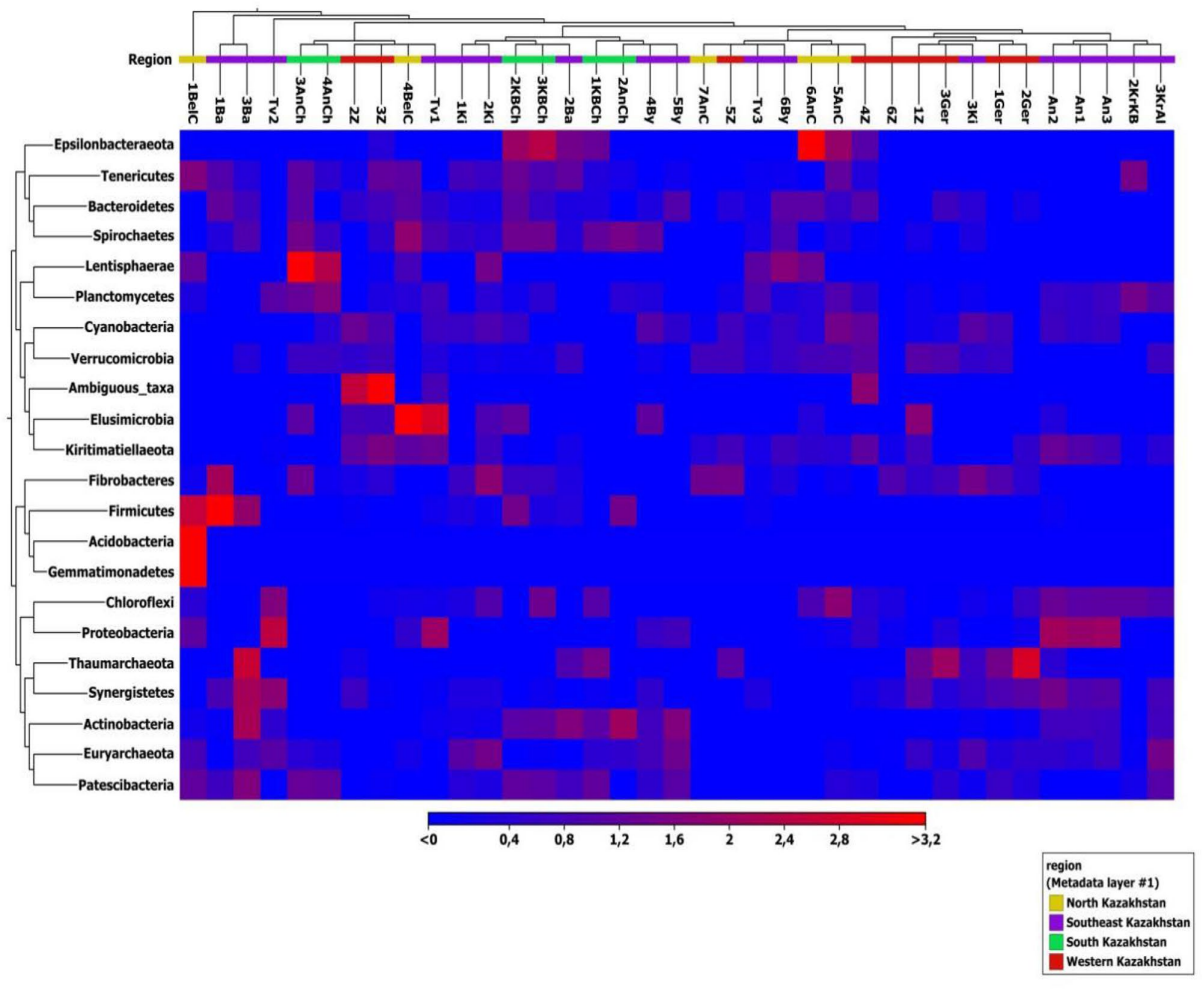
Heat-map of microbial community composition with cluster analysis. The color intensity in each panel shows the relative abundance in a sample, referring to color key at the bottom.

To visualize the differences in the bacterial community structures of the samples, a heat-map with cluster analysis was constructed at the phylum level (Fig. 4).

When developing a heat map, twenty-two phyla from four regions of Kazakhstan were taken into account. Figure 4 shows that the most numerous in all samples were *Firmicutes* (57.3%). The dominant phyla were *Bacteroidetes* (17.0%), *Verrucomicrobia* (6.88%), *Euryarchaeota* (6.49%), *Actinobacteria* (4.77%) and *Patescibacteria* (3.38%).

Further, diversity indices of fecal bacterial communities in different regions were determined.

Rarefaction curves showing the magnitude of the detected OTUs depending on the selective effort did not reach a plateau in all samples of the study (Supplementary figure S1), probably also for the type of samples analyzed: optimal amplification with fecal samples was not always possible. Nevertheless, to allow a comparison among all the samples, a sequencing depth of 13,000 reads was selected for the comparisons, in order to obtain a complete overview.

**Figure 5:**
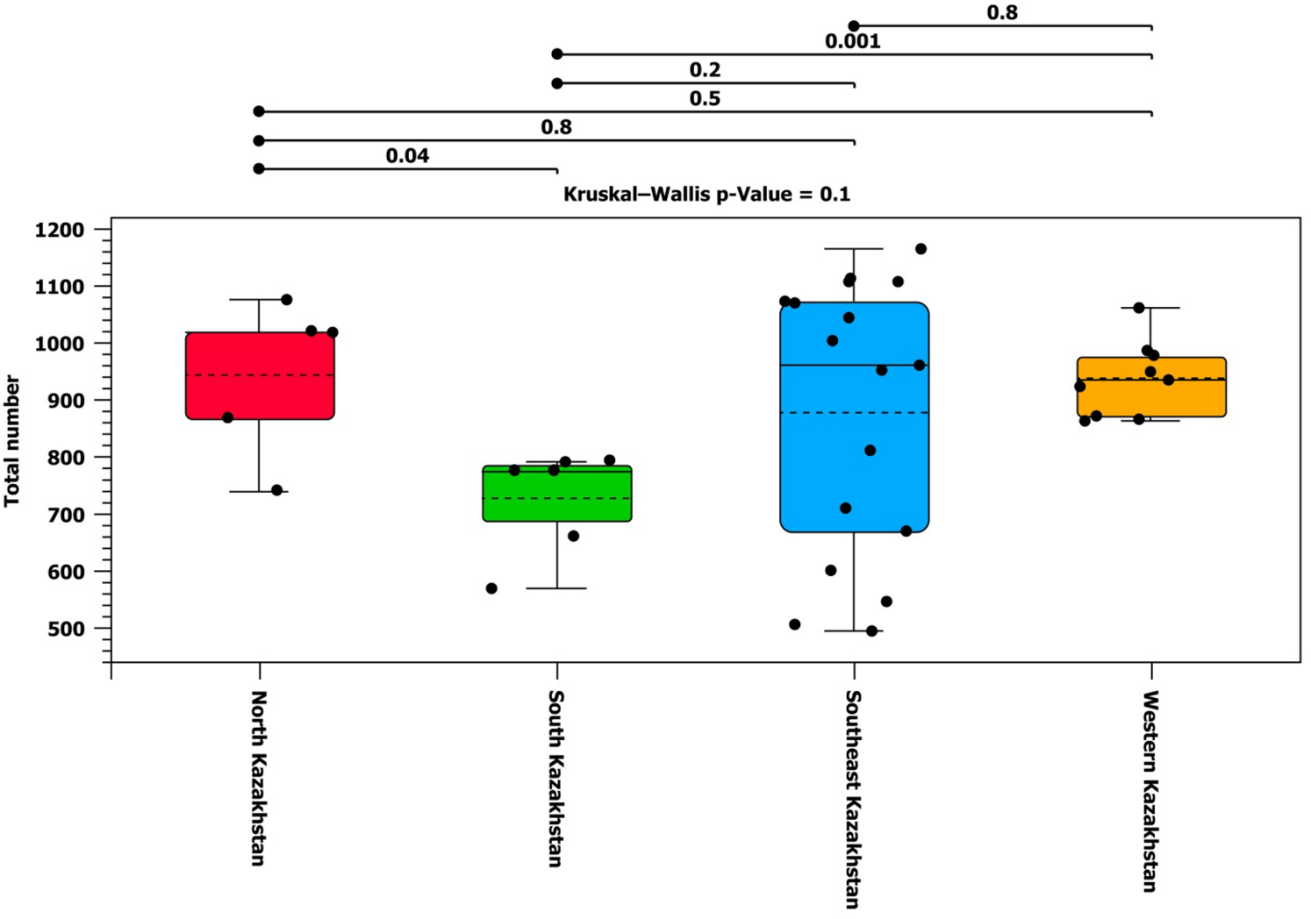
Box plots of alpha diversity by Kazakh region calculated from the set of OTU abundances.

**Figure 6:**
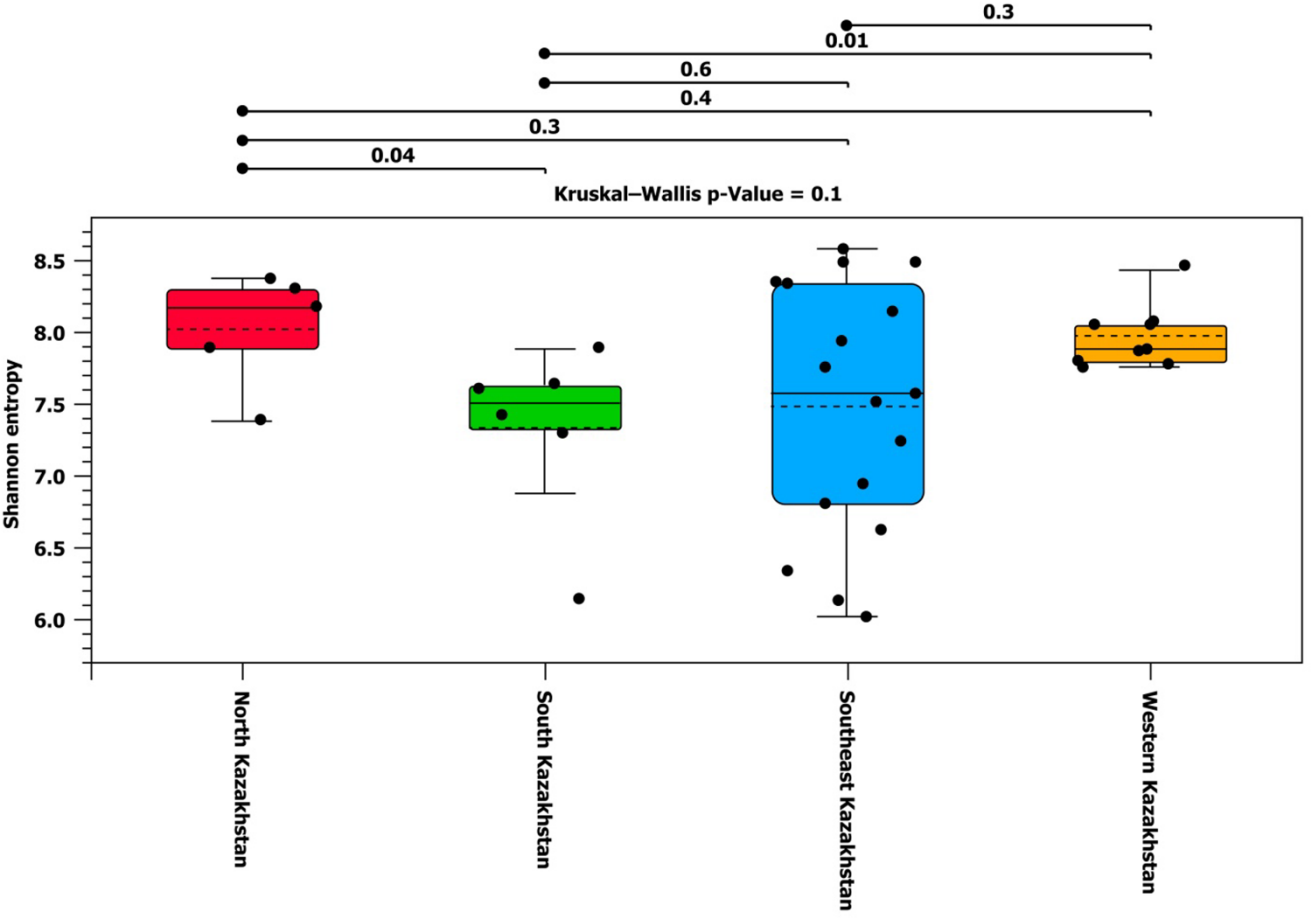
Alpha diversity of Kazakhstan regions using the Shannon index calculated from the set of OTU abundances.

To assess alpha diversity, the species richness index and the Shannon index were calculated (Figs. 5,6). Shannon diversity accounts for both abundance and evenness of taxa present. Figures 5 and 6 showed the diversity of samples across the Kazakhstan regions.

Alpha diversity indices, including the species richness index and the Shannon index, were calculated and analyzed using the Wilcoxon rank sum test between the two groups to determine the p-value for group comparisons. It was found that there is a significant difference between the variability of microbiota of cattle from North and South Kazakhstan (p-value: 0.04) and between South and West Kazakhstan (p-values: 0.001 and 0.01).

**Figure 7:**
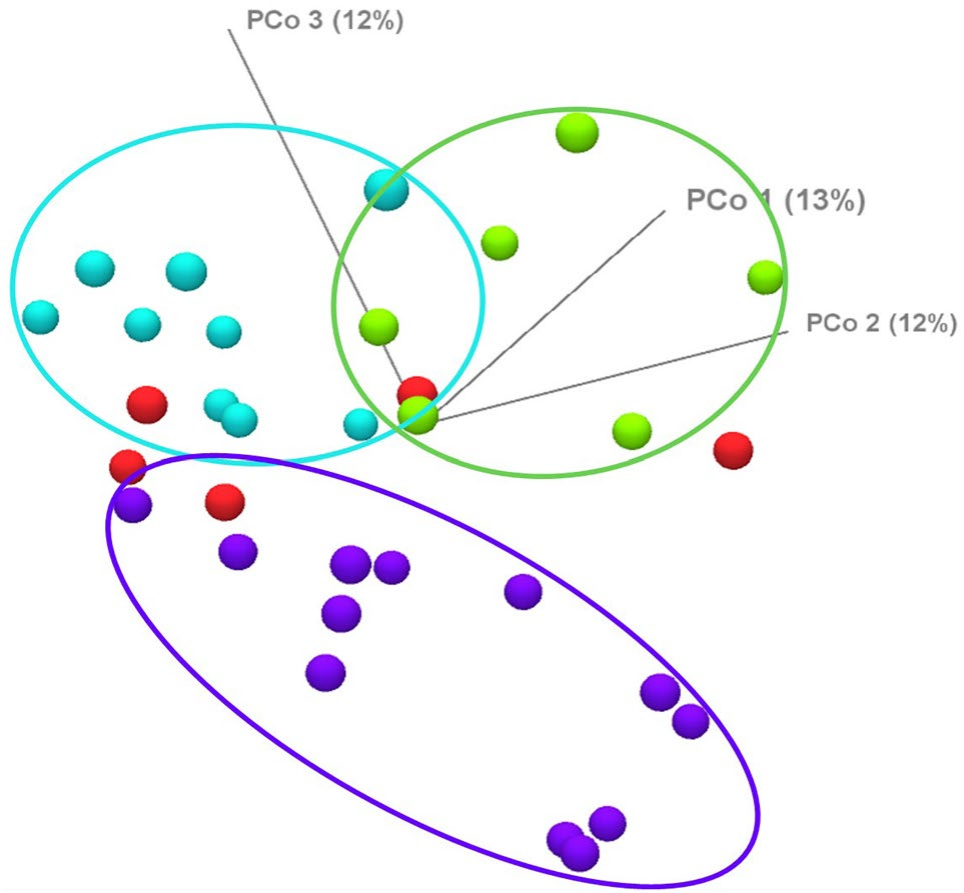
The effect of the regions on beta diversity of fecal bacterial community as shown by principal coordinate analysis (PCoA) based on Bray–Curtis index (green dot = South Kazakhstan; purple dot = Southeast Kazakhstan; red dot = North Kazakhstan; light blue dot = Western Kazakhstan). PCoA was carried out starting from the set of OTU abundances. Regional clusters which resulted significantly different by PERMANOVA analysis are highlighted by ovals.

Beta diversity examines the change in species diversity between microbiotas. Principal coordinate analysis (PCoA) was performed to examine the beta diversity based on Bray–Curtis index (Fig. 7).

The group separation observed on PCoA was further tested for significance by Permutational multivariate analysis of variance (PERMANOVA).

Table 3 shows the metadata variable used, its groups and the results of the test (pseudo-f-statistic and p-value), which resulted in a significant difference between groups.

**Table 3:** Beta diversity by Kazakh region.

Variable	Groups	Pseudo—f—statistic	p-value
Region	South Kazakhstan, Southeast Kazakhstan, Western Kazakhstan, Northern Kazakhstan	2,20,100	0,00,002

Table 4 shows the PERMANOVA analysis for each pair of groups and the results of the test (pseudo-f-statistic and p-value). Bonferroni-corrected p-values are also shown.

**Table 4:** PERMANOVA analysis in comparison of regions.

Group 1	Group 2	Pseudo—f—statistic	p-value	p-value (Bonferroni)
South Kazakhstan	Southeast Kazakhstan	1.87495	0.01325	0.07950
South Kazakhstan	Northern Kazakhstan	1.73739	0.01515	0.09091
Southeast Kazakhstan	Northern Kazakhstan	1.54289	0.05411	0.32468
South Kazakhstan	Western Kazakhstan	3.04263	0.00100	0.00599
Southeast Kazakhstan	Western Kazakhstan	2.87394	0.00014	0.00084
Northern Kazakhstan	Western Kazakhstan	2.19016	0.01798	0.10789

Table 4 shows that two Bonferroni p-values maintain significance after Bonferroni-correction, that is when comparing samples from Western Kazakhstan with Southern (p-value 0.00599), and samples from Southeast Kazakhstan with Western Kazakhstan (p-value 0.00084). Thus, Western Kazakhstan differs from other areas of the countries in terms of beta diversity.

## Discussion

In our experience, we studied fecal samples from 37 heads of animals belonging to meat and dual-purpose breeds from different regions of Kazakhstan by 16S metabarcoding analysis.

As a result of bioinformatic analysis, it was revealed that the most common phylum of bacteria was *Firmicutes* in the Northern (61.3%), Western (60.5%), where mainly hay and pasture types of feeding were used. In the Southern and Southeast (55.5%) regions of Kazakhstan with the hay-concentrate type of feeding. Next in terms of prevalence is the phylum of *Bacteroidetes* in the Northern (18.7%), Western (20.4%), Southern (15.3%) and Southeast (15.7%) regions of Kazakhstan.

The dominant phyla are consistent with previous studies of bovine intestinal bacteria, in which the Firmicutes phylum is the most abundant, followed by *Bacteroidetes* and *Proteobacteria*^13,14^. In the South-Eastern region of Kazakhstan, cattle are fed a large amount of grain, in connection with this, the relative abundance of representatives of *Proteobacteria* types in the microbial community in the digestive tract increases, which leads to a decrease in the proportion of *Firmicutes.*

The composition of the microbial community depends on the diet. Previously, the relationship between diet and microbiota was described: Mao et al.^15^ and Li et al.^16^ showed that feeding a large amount of grain increased the relative abundance of *Firmicutes* and decreased *Bacteroidetes* and *Fibrobacter* in the microbial community of the bovine digestive tract livestock and increased the ratio between *Firmicutes* and *Bacteroidetes*, as a result of which *Firmicutes* and other opportunistic types, such as *Proteobacteria*, reproduce faster per unit time ^17^. However, other author had opposite results. Animals fed with hay had more bacteria belonging to the *Fibrobacteres* type, and those fed with grain, more bacteria belonging to the type *Bacteroidetes* ^17–19^.

A decrease in the relative abundance of *Bacteroidetes* and *Fibrobacteres* in the rumen microbial community leads to subacute rumen acidosis^17^.

El Kaoutari et al. suggested that members of *Bacteroidetes* contain a large amount of glycoside hydrolase (GH) and polysaccharide lyase (PL) enzymes; therefore, they are the primary destructors of complex polysaccharides in the plant cell wall. In this regard, an increase in the ratio of *Firmicutes* to *Bacteroidetes* is harmful for the animal^20^.

Martinez-Fernandez et al. described that animals fed with hay showed a more efficient redirection of H_2_ to other microbial products compared to animals fed with the hay-concentrate diet^21^.

Feeding animals with hay increases the ratio of *Bacteroidetes* and *Firmicutes* and decreases *Archaea* and *Synergistetes* in the liquid phase of the rumen content.

*Bacteroidetes *are considered to be H_2_ users, while *Firmicutes* produce H_2_^21^.

Methanogenic archaea are a large and diverse group of microorganisms. They have three important properties in common: they all produce methane, are strict anaerobes, and belong to the archaea of the *Euryarchaeota* type. Methane-forming archaea play a key role in the anaerobic decomposition of organic matter in the absence of acceptors such as nitrates, sulfates, Fe (III), and Mn (IV), which maintain a higher redox potential in the environment. These microorganisms are involved in the formation of biogenic methane in their habitats. In our study, the methanogens of *Euryarchaeota* were less abundant in animals from the Western region (2.4%), and most present in animals from the Southeastern region (9.6%). The largest number of members of the order *Methanobacteriales* was found in Southeast Kazakhstan, while they were less represented in Western Kazakhstan. *Methanobrevibacter* is present in more than 80% of 16S rRNA genes, sequenced from birds and various animal species^22^. We found the highest amount of *Methanobrevibacter* in the Southeast region of Kazakhstan (8.6%), and the lowest in the Western region (1.8%). With regard to other methanogens genera, *Methanosphaera* was relatively more present in the Southeast region (0.65%), while decreasing to 0.13% in the South region. Other representatives of the genus of methanogens were present in traces.

In the beef cattle feeding industry, the grain-fed method feeds young stock in the feedlots with a high grain diet, the grass-fed regime allows livestock to naturally graze on pastures using grass-based diets. As the demand for healthy and appetizing beef grows worldwide, grass-fed beef products are gradually gaining more and more attention in the meat market; a growing group of consumers are willing to pay a higher price for grass-fed beef than for grain-fed beef^14^. Significantly higher levels of microbial diversity have been reported in the faeces of grass-fed cattle compared to grain-fed cattle^14^. Our results are consistent with the data of these authors. Western Kazakhstan differs from other regions in beta diversity. This is probably due to the fact that the animals grazed in the pasture. The data are consistent with findings by others who have found that improved pasture quality can improve dietary digestibility and lead to reduced intestinal CH_4_ emissions^23–25^. Despite a relatively high share of greenhouse gases from livestock production, grazing systems are associated with lower greenhouse gas emissions than other agricultural systems^26,27^. Animals grazing in the Western Region had quality pasture with tall and dense grass. Animals grazing in the South-East region had poor quality pasture with sparse vegetation, due to the fact that there was a drought in the region. In this regard, the amount of methane was released more in the animals of the South-Eastern region.

The alpha and beta diversity showed high variability for samples from Southeast Kazakhstan. Actually, this group was larger than the others (17 animals), and characterized by different breeds and feeding types. These conditions can influence the variability of the group and may explain its higher level, as compared to the other three groups.

According to alpha diversity indices, there are significant differences between the microbiota of cattle from North and South Kazakhstan, and between South and Western Kazakhstan. Thus, the samples from North Kazakhstan have a more diverse microbiota compared to the rest of the country. This finding is likely due to the fact that animals were imported from Ireland.

Western Kazakhstan differs from other regions with regard to beta diversity. This is probably due to the fact that animals were grazed in the pasture. Thus, grazing animals leads to a decrease in methane emissions.

## Conclusions

Methanogens belonging to *Euryarchaeota* phylum*,* order *Methanobacteriales*, genus *Methanosphaera* were less abundant in animals from the Western region, and most present in animals from the Southeastern region.

There are significant differences between the microbiota of cattle from North and South Kazakhstan, and between South and Western Kazakhstan. Thus, the samples from North Kazakhstan have a more diverse microbiota compared to the rest of the country. The difference between the Western and Southern regions is explained by the different type of feeding.

Western Kazakhstan differs from other regions with regard to beta diversity. This is probably due to the fact that animals are grazed in the pasture. Thus, grazing animals leads to a decrease in methane emissions.

## Supplementary Information

Supplementary Information.

## Data Availability

The data presented in this study are publicly available at the NCBI Sequence Read Archive (Bioproject ID: PRJNA847733).

## Abbreviations

CH_4_: Methane
GHG: Greenhouse gas
OTU: Operational taxonomic unit
PCoA: Principal coordinates analysis
PERMANOVA: Permutational multivariate analysis of variance
