# Supplementary material for: Study of cattle microbiota in different regions of Kazakhstan using 16S metabarcoding analysis
Source: Sci Rep. 2022 Sep 30;12:16410. doi: 10.1038/s41598-022-20732-4 (PMC9525287; doi:10.1038/s41598-022-20732-4)
Supplement: Supplementary file 1 — Supplementary Information. [file 41598_2022_20732_MOESM1_ESM.pdf]

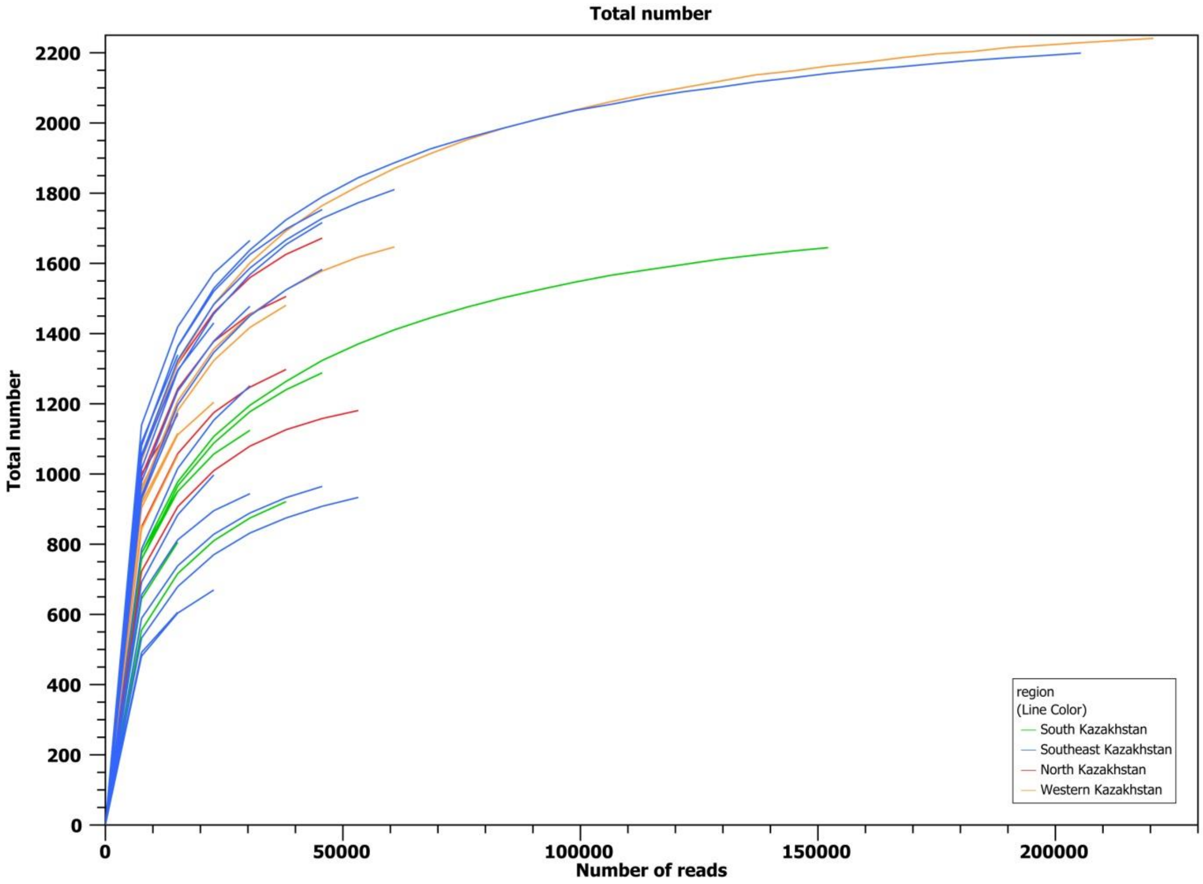

Supplementary figure S1. Rarefaction curves plotted for the analyzed microbiotas. The abscissa shows the number of sequences in the sample. The ordinate indicates the total number of taxa at the genus level.
